# Strain-encoding cardiovascular magnetic resonance for assessment of right-ventricular regional function

**DOI:** 10.1186/1532-429X-10-33

**Published:** 2008-07-04

**Authors:** Amr Youssef, El-Sayed H Ibrahim, Grigorios Korosoglou, M Roselle Abraham, Robert G Weiss, Nael F Osman

**Affiliations:** 1 Department of Medicine, Division of Cardiology, Johns Hopkins University, Baltimore, MD, USA; 2 Cardiology department, Ain Shams University, Cairo, Egypt; 3 Department of Radiology, University of Florida, Jacksonville, FL, USA; 4 Russell H. Morgan Department of Radiology and Radiological Science, Johns Hopkins University, Baltimore, MD, USA; 5 University of Heidelberg, Department of Cardiology, Heidelberg, Germany; 6 Department of Electrical and Computer Engineering, Johns Hopkins University, Baltimore, MD, USA

## Abstract

**Background:**

Tissue tagging by cardiovascular magnetic resonance (CMR) is a comprehensive method for the assessment of cardiac regional function. However, imaging the right ventricle (RV) using this technique is problematic due to the thin wall of the RV relative to tag spacing which limits assessment of regional function using conventional in-plane tagging.

**Hypothesis:**

We hypothesize that the use of through-plane tags in the strain-encoding (SENC) CMR technique would result in reproducible measurements of the RV regional function due to the high image quality and spatial resolution possible with SENC.

**Aim:**

To test the intra- and inter-observer variabilities of RV peak systolic strain measurements with SENC CMR for assessment of RV regional function (systolic strain) in healthy volunteers.

**Methods:**

Healthy volunteers (n = 21) were imaged using SENC. A four-chamber view was acquired in a single breath-hold. Circumferential strain was measured during systole at six equidistant points along the RV free wall. Peak contraction is defined as the maximum value of circumferential strain averaged from the six points, and regional function is defined as the strain value at each point at the time of peak contraction.

**Results:**

Mean values for peak circumferential strain (± standard deviation) of the basal, mid, and apical regions of the RV free wall were -20.4 ± 2.9%, -18.8 ± 3.9%, and -16.5 ± 5.7%, Altman plots showed good intra- and inter-observer agreements with mean difference of 0.11% and 0.32% and limits of agreement of -4.038 to 4.174 and -4.903 to 5.836, respectively.

**Conclusion:**

SENC CMR allows for rapid quantification of RV regional function with low intra- and inter-observer variabilities, which could permit accurate quantification of regional strain in patients with RV dysfunction.

## Background

Despite the rapid advances in the field of cardiovascular imaging over the past decade, assessment of the right ventricular (RV) function is still challenging [1]. However, accurate assessment of RV function is an important predictor of clinical outcome in patients with congenital heart disease, pulmonary hypertension [2,3], myocardial infarction [4,5], heart failure [6], dilated cardiomyopathy [7], and arrhythmiogenic RV dysplasia (ARVD) [8].

Cardiovascular Magnetic Resonance (CMR) with its superior tissue contrast, high spatial and temporal resolution, and non-invasive nature is an important modality for assessment of global RV function [9]. By imaging the induced magnetization of different tissues, and not just their anatomic borders, CMR provides greater flexibility in imaging the structure and function of the heart. Specifically, in CMR tagging, parallel planes of saturated magnetization can be applied orthogonal to the imaging plane, which create non-invasive MR-visible markers, or tags, that move with the contraction of the heart [10,11] and permit quantification of its strain [12] in the three-coordinate directions (circumferential, longitudinal, and radial). With advances in signal processing, the amount of displacement of these tags within the myocardium can be automatically quantified using harmonic phase (HARP) analysis, which allows for rapid and accurate analysis of myocardial regional function [13]. CMR tagging is considered the gold standard technique for assessment of left ventricular (LV) regional function [14]. However, the thin wall of the RV (normally < 5 mm) results in tags those are too far apart for accurate assessment of thin walled RV regional function.

In this work, we propose to use the through-plane tags in strain encoding (SENC) CMR [15] in order to overcome the limitations posed by the thin-walled RV and to allow for quantitative assessment of the RV regional function. SENC CMR has several advantages over regular tagging: 1) Spatial and temporal resolution are improved. 2) The signal intensity depends on the spacing of the invisible tags in the through-plane direction, which allows for strain calculation in the direction perpendicular to the imaging plane [16]. Because of the latter, circumferential strain can be measured from the long-axis view, while the presence of the tags does not affect visualization of different cardiac structures. 3) The circumferential strain of the RV free wall can be measured from a single four-chamber view instead of multiple short-axis views in conventional tagging. Despite these theoretical advantages, the value of SENC CMR for studying human RV function has not been investigated thoroughly. In this study, the intra- and inter-observer variabilities of SENC CMR were evaluated for assessment of RV regional function in healthy volunteers.

## Methods

The study was approved by the Institutional Review Board of Johns Hopkins Medical Institutions, and informed consent was obtained from all participants prior to the MR exam. The study was Health Insurance Portability and Accountability Act (HIPAA) compliant.

### Volunteer selection

Healthy volunteers (n = 21) with no history of heart disease and no abnormal cardiac structure or function were studied by CMR.

### SENC CMR

**Figure 1:**
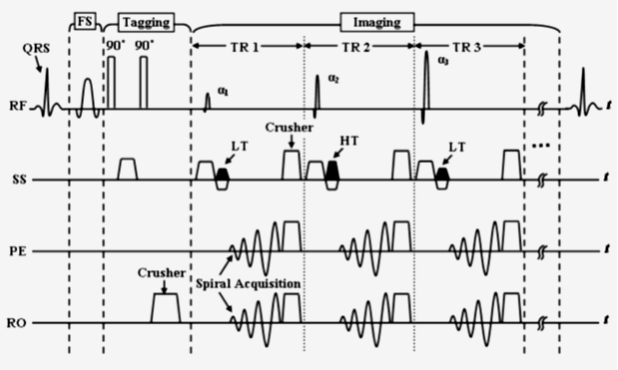
SENC pulse sequence diagram (FS: fat suppression, RF: radiofrequency, SS: slice selection, PE: phase encoding, RO: readout, HT: high-tuning, LT: low-tuning).

SENC CMR is based on the acquisition of two images with different frequency modulation, or "tunings", in the slice-selection direction. We call these images the low-tuning (LT) and high-tuning (HT) images. Bright regions in the LT and HT images represent static and contracting tissues, respectively [16]. The two SENC images were combined as described in [16] to result in a strain image, where signal intensity is proportional to through-plane strain. Strain measurements calculated from SENC images have recently been validated against standard Spatial Modulation of Magnetization (SPAMM) tagged images [17,18]. Figure 1 shows a diagram of the SENC pulse sequence [16]. It consists of two major modules: tagging and imaging. The tagging part is implemented after the detection of the R-wave of the electrocardiogram (ECG), and it is composed of two 90° non-selective radiofrequency (RF) pulses interspersed by a modulation gradient in the slice-selection direction and followed by crusher gradients in all directions. A spectral-selective fat suppression RF pulse was applied immediately before the tagging module [19]. Imaging started directly after the tagging module, where a series of slice-selective RF pulses were applied with alternating SENC modulations (tunings) and ramped flip angles [20]. K-space was filled in a segmented fashion with spiral acquisition for efficient readout.

### CMR scan protocol

Studies were performed on a 3.0T MR scanner (Philips Medical Systems, Best, The Netherlands) using a 6-element phased-array cardiac coil. Four ECG leads were placed on the volunteers' chests for triggering the pulse sequence at the R-wave of the ECG. The volunteers' position was head first and supine. After initial scouting images and reference scan, gradient echo vertical long axis cine (pseudo two-chamber view) images were obtained from the coronal images by aligning the left ventricular apex with the center of the mitral valve. From the end-systolic vertical long-axis image, an initial horizontal long-axis plane (pseudo four-chamber view) was obtained by aligning through the apex and the midpoint of the mitral valve. Then, a single short-axis plane was obtained from the end-systolic horizontal long axis image. The final four-chamber view was obtained from the-short axis plane by bisecting both ventricles and going parallel to the diaphragm. The measurement points on the SPAMM and SENC images were determined with the help of the geometry scouting images, in order to assure that the measurement positions in the SPAMM images correspond to their counterparts in the SENC images.

The imaging parameters for the gradient echo cine acquisitions were: repetition time (TR) = 5 ms; echo time (TE) = 2.9 ms; flip angle = 15°; scan matrix = 176 × 138 reconstructed to 256 × 256 pixels; field of view (FOV) = 35 × 35 cm; and slice thickness = 8 mm. Four-chamber SENC images were then acquired during a single breath-hold of 13 heartbeats using prospective ECG gating. SENC imaging parameters were similar to those of the cine images, except for: spiral acquisition (for efficient signal readout) with 12 spirals × 12 ms; scan matrix = 176 × 176; TR = 14.4 ms; TE = 0.8 ms; flip angle = 40°; and slice thickness = 8 mm. For SENC images, the temporal resolution was 15 msec and the spatial resolution was 2 × 2 × 8 mm^3^. Finally, dynamic short-axis SPAMM grid-tagged images were acquired at the same locations as the cine images, and with similar imaging parameters, except for: TR = 12 ms; TE = 1.8 ms and flip angle = 35°.

### CMR data analysis

**Figure 2:**
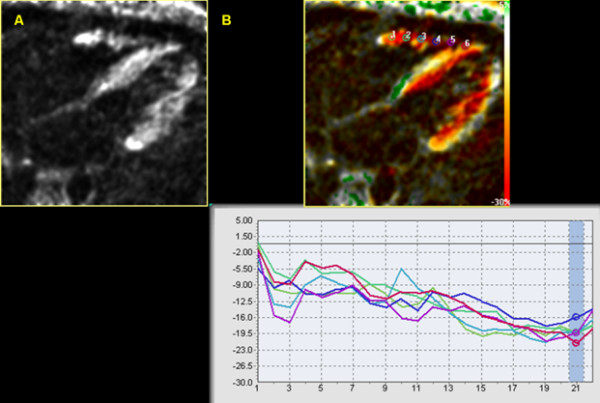
**Four-chamber SENC CMR view at the end-systolic frame.** A) The RV as appears on the acquired image before software processing. B) The processed image showing the position of the six points along the RV free wall and the corresponding strain curve. Y-axis shows the strain values while the X-axis shows the time frames.

Tagging and SENC CMR data were analyzed using the HARP and SENC software packages (Diagnosoft Inc., Palo Alto, California), respectively. For each four-chamber SENC image (Figure 2), six points were manually selected (by observer AY) along the RV free wall in the end systolic frame, starting from the base toward the apex with equal distances from each other. Systolic circumferential strain was calculated at each of the six points on the SENC image, as described in [16]. Strain estimates were also computed from the short-axis SPAMM tagged images, as described in [13]. Myocardial strain was defined here as the percentage change in tissue length from the resting state at end-diastole (ED) to the one achieved following myocardial contraction at end-systole (ES): Myocardial strain = (ED - ES)/ED × 100%.

In order to detect the timing of end-systole in the SENC cine, the time point of maximum average strain was selected to represent end-systole with the peak systolic strain is the stain estimate recorded at this time point as shown in figure 2B at time frame 21.

The SENC derived myocardial strain values were calculated by the same observer (AY) two weeks after the first reading to report the intra-observer variability. The strain values were also calculated by a second independent observer (GK), blinded to the results obtained by the first observer, to determine inter-observer variability. SENC and SPAMM strain estimates of the interventricular septum were calculated by the first observer, using the same method described above, to serve as a reference for the RV free wall strain estimates.

Strain noise was then calculated for the RV free wall in the 21 SENC CMR scans. This was done by quantifying the noise in the strain estimate by examining a small region around points within the base, mid and apical RV free wall and reporting the standard deviation in the strain estimate at those points. The standard deviation in the strain estimates were divided by the mean strain estimates at those regions. Then, SENC strain estimate was correlated with strain noise. A subgroup of SENC CMR scans with the least strain noise and absence of image artifacts were then selected. The purpose of this sub-grouping was to examine the effect of image quality on the intra- and inter observer variabilities of the strain estimates.

### Statistical analysis

Statistical analysis was performed using commercially available software (STATA, version 9.2, College Station, Texas). Strain estimates were presented as mean ± standard deviation (SD). Circumferential strain estimates were compared using a paired t-test for normally distributed data, Wilcoxon signed rank sum test for non-normally distributed data. Multiple measures analysis of variance (ANOVA) analysis was used to compare the average circumferential strain measurements of the base, mid, and apical parts of the RV free wall. *P *< 0.05 was considered statistically significant.

Intra- and inter-observer agreements were evaluated using the 95% limits-of-agreement approach proposed by Bland and Altman [21], by plotting the differences between each pair of measurements against their means. "Limits of agreement" was presented as mean difference ± 2 SD. Assuming normal distribution, it is expected that most differences (>95%) would lie between mean - 2SD and mean + 2SD (limits of agreement). To test if the variances of strain estimates were the same within the same observer and between observers, Pitman's test [22] for correlated variances was used. Also, linear regression analysis was performed between the end-systolic circumferential strains of the RV free wall measured by the first observer two weeks apart, and between the first and second observers. Intra-observer and inter-observer variabilities were calculated from the linear regression analysis using the intra-class correlation coefficient (r).

## Results

Twenty-one healthy volunteers with ages ranging from 23 to 45 years (mean age of 35 ± 7 years standard deviation) were included in the study. All the scans were done at Johns Hopkins University during the year 2006. The average scanning time was 35 minutes. All the images acquired from the twenty-one studies were suitable for analysis. The contracting RV gives bright signal on the four-chamber SENC CMR against a dark background (Figure 2). The acquired images were then processed for strain analysis using the SENC software.

### The peak circumferential systolic strain estimates of RV free wall and interventricular septum

**Figure 3:**
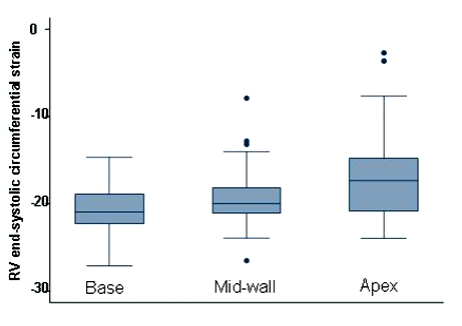
**Box-and-whisker plots of peak SENC strain estimates for the base, mid-wall and apex of the RV free wall. **The whiskers are drawn from the box to the highest and lowest values that are within 1.5 times the interquartile range of the median with any points more extreme than this are plotted individually.

**Figure 4:**
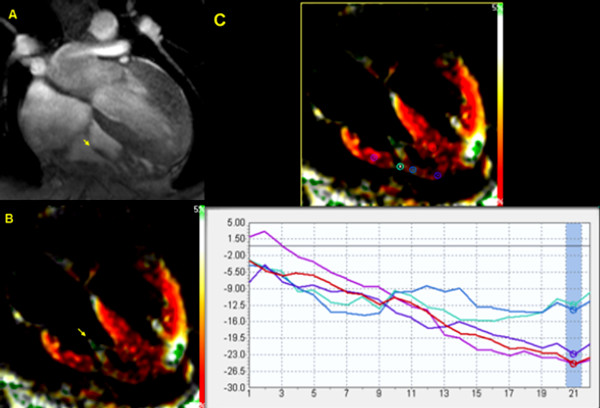
**Site of attachment of the papillary muscle into the RV free wall shows area of low strain as compared to the rest of the RV free wall.** A) Gradient echo image with the arrow pointing to the papillary muscle. B) Same view on SENC CMR. C) SENC strain analysis showing area of low strain estimate on RV free wall corresponding with the site of attachment of papillary muscle.

The average peak circumferential strain of the RV free wall measured by SENC CMR was -18.7 ± 4.3% (the minus sign means positive contraction). The average strain ± standard deviation for the basal, mid, and apical regions of the RV free wall were -20.7 ± 2.8%, -19.1 ± 3.3% and -16.9 ± 4.8%, respectively (Table 1 and Figure 3). There is a statistically significant difference in the SENC circumferential systolic strain estimates among the base, mid and apical segments of the RV free wall (P = 0.0015). Myocardial areas of consistent low circumferential strain were found in 8 out of the 21 scans close to the site of insertion of the papillary muscle into the RV free wall (Figure 4).

**Table 1:** The average values for SENC peak circumferential systolic strain estimates of the RV free wall.

**Region/Observer**	**Average SENC peak systolic strain estimate (± SD) measured by the 1st observer**	**Average SENC peak systolic strain estimate (± SD) measured by the 1st observer (second time)**	**Average SENC peak systolic strain estimate (± SD) measured by the second observer**	**Average SPAMM peak systolic strain estimate (± SD) *(P value)***
**Basal segment of RV free wall**	-20.7% (± 2.8)	-20.4% (± 2.8)	-19.9% (± 2.9)	-18.8% (± 2.5) *(P = 0.03)*
**Middle segment of RV free wall**	-19.1% (± 3.3)	-19.0% (± 3.4)	-19.6% (± 3.8)	-18.5% (± 2.7) *(P = 0.35)*
**Apical segment of RV free wall**	-16.9% (± 4.8)	-16.7% (± 5.1)	-17.8% (± 4.6)	-18.3% (± 3) *(P = 0.08)*

The average RV free wall circumferential strain measured from the short-axis SPAMM tagged slices in the same subjects was -18.5 ± 2.7% (P = 0.42). The SPAMM circumferential strain estimates for the base, mid, and apical slices of the RV free wall were -18.8 ± 2.5% (P = 0.03), -18.5 ± 2.7% (P = 0.35) and -18.3 ± 3.0% (P = 0.08), respectively. There is no statistically significant difference in the SPAMM circumferential systolic strain estimates between the base, mid and apical segments of the RV free wall (P = 0.67).

The average value of the circumferential peak systolic strain estimate of the interventricular septum was -21.2 ± 3.2% as measured by SENC CMR in four-chamber view and -20.6 ± 2% (P = 0.14) as measured by HARP analysis of grid tagging in short-axis views. The SENC circumferential strain estimates for the base, mid, and apical slices of the interventricular septum were -20.5 ± 2.7%, -21.7 ± 3.1% and -20.3 ± 3.0%, respectively. Compared with SENC circumferential systolic strain estimates of the RV free wall, there was no significant difference between the basal segments (P = 0.13), while mid and apical segments of the RV free wall were significantly less (P < 0.01) than those of the interventricular septum.

### Intra-observer and inter-observer agreements

**Figure 5:**
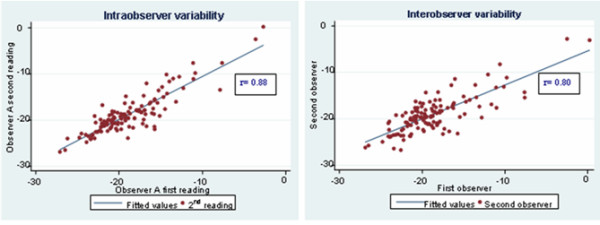
Intra and inter-observer variabilities as indicated by interclass correlation co-efficient (r) on linear regression analysis.

Regional peak circumferential systolic strain measurements demonstrate low intra- and inter-observer variabilities (Figure 5) as indicated by the interclass correlation coefficient (r = 0.82/0.81; 0.80/0.79; 0.94/0.81 for the basal, mid, and apical regions, respectively). The overall intra- and inter-observer variabilities were r = 0.88 and 0.80, respectively.

**Figure 6:**
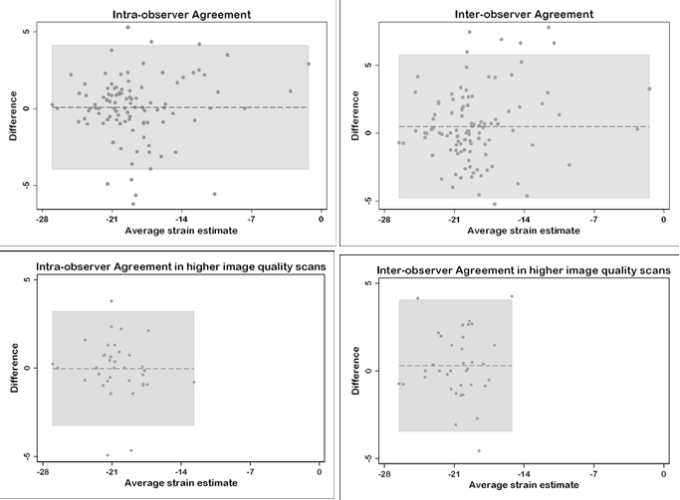
**Bland-Altman plots for intra-observer and inter-observer agreements.** Higher image quality scans showed higher degree of agreements. In all plots, the dashed lines represent the mean difference and the gray shaded areas represent the limits of agreements.

Bland Altman plots have shown the mean difference between the SENC strain estimates obtained by the same observer to be 0.11% (95% confidence interval = -0.24% to 0.46%) and between the two observers to be 0.32% (95% confidence interval = -0.14 to 0.79%). The intra-observer and inter-observer limits of agreement were -4.038 to 4.174 and -4.903 to 5.836, respectively (Figure 6). There was no significant intra-observer or inter-observer difference in variances (Pitman's coefficient [r] = 0.1, P = 0.28; and [r] = 0.14, P = 0.13, respectively).

### Effect of image quality on strain estimates, intra- and inter-observer agreements

The SENC average peak circumferential systolic strain estimates from the group of subjects with highest image quality was 1.8% (-20.5 ± 2.8% versus -18.7 ± 4.3%, P < 0.02) higher than the average strain estimates. Also, there was slightly higher agreement among the group of highest image quality scans than the average agreement on Bland Altman plots with a mean intra-observer difference of 0.027% (95% confidence interval = -0.53% to 0.58%) and inter-observer difference of 0.30% (95% confidence interval = -0.35 to 0.94) and limits of agreement of -3.3 to 3.3 and -3.5 to 4.1 for the intra-observer and inter-observer, respectively (Figure 6).

## Discussion

This study demonstrates that circumferential strain of the RV free wall can be obtained using SENC CMR with low intra- and inter-observer variabilities. The average RV free wall circumferential strain estimate with SENC (-18.9 ± 4.1%) was in good agreement with previous results obtained by CMR tagging [23] using one-dimensional SPAMM tagging. In that study, the RV free wall average circumferential strain in normal volunteers ranged from -16.4 ± 1% in the RV outflow tract to -22.4 ± 1.5% at the RV apex. However, the lower strain values of the RV apex in our study could be attributed to 1) the method of selecting the points for strain analysis from areas with low signal (e.g., around the insertion site of the papillary muscle); and/or 2) the weaker signal captured from the thin walled apex in low image quality studies, which resulted in abnormally low strain values, shown as outliers in Fig. 3. In our study, the SPAMM grid-tagged circumferential systolic strain estimates from the apical portion of the RV free wall was -1.4% higher than the SENC strain estimates, but this difference did not reach statistical significance (*P *= 0.08). With not much difference in the strain estimates between SENC and SPAMM grid tagging for the RV free wall, the major advantages of SENC are 1) obtaining the entire strain data from a single view rather than 3 short-axis views in grid tagging; 2) better visualization of the contracting myocardium which allows for more accurate localization of the region of interest; and 3) the resulting strain curves are interpretable from the entire RV free wall.

To be more confident about our strain estimates and to avoid any systematic bias, we used both SENC and SPAMM grid-tagging circumferential strain estimates of the interventricular septum, where excellent strain curves were obtained in almost all subjects, as a reference. There was no significant difference in the circumferential strain estimates of the interventricular septum between the methods (-21.2 ± 3.2% for SENC CMR versus -20.6 ± 2% for SPAMM grid tagging (P = 0.14)). Also, there was no significant difference between the basal segments of RV free wall and interventricular septum (P = 0.13). However, the circumferential strain estimates of the mid and apical segments of the RV free wall were significantly less (P < 0.01) than those of the interventricular septum. This could be attributed to the lower strain SNR of apical regions in images with marginal quality or the attachment site of the papillary muscle into the RV free wall in other images.

The completely automatic calculation of RV strain values with SENC produces good intra-observer and inter-observer agreement. This was particularly evident in the high-quality images where there is less noise to confound the actual strain results.

The effect of image quality on circumferential myocardial strain quantification was also addressed in our study. The average RV circumferential strain measured from the group of scans with the least strain noise and image artifact was found to be statistically significant higher than the average strain estimate of the 21 studies (-20.5 ± 2.8% versus -18.7 ± 4.3%, P < 0.02). Also, plotting the SENC strain estimates against noise in the strain estimates revealed a significant linear relation. It should be noted that SENC imaging is one type of STEAM (stimulated-echo acquisition mode) pulse sequence, which suffers from 50% signal loss, making SENC imaging sensitive to image SNR.

Circumferential strain measurements were conducted in this study based on the observation that myocardial contraction is principally circumferential [24]. On the contrary, tissue Doppler data suggested that RV contraction is mainly longitudinal [25]. However, tissue Doppler could only reliably calculate the longitudinal functional parameters of the RV due to its thin wall [26]. Another study of the RV strain in open-chest, open-pericardium animal models [27] showed that the RV longitudinal strain correlates with the RV global function during both basal conditions and following an increase in afterload, whereas circumferential strain is influenced by changes in afterload. This could make circumferential strain analysis even more specific and clinically useful in pressure overload conditions, like pulmonary hypertension, which constitute the majority of RV pathologies.

Overall, it should be noted that the use of spiral acquisition for data readout, automated software for strain analysis and acquisition of circumferential strain estimates for the entire RV from a single four-chamber view made the entire process time-efficient and practical.

### Study limitations and future directions

As this study was conducted only on normal volunteers, one limitation is that the diagnostic utility of the strain measurements were not tested on patients with known RV dysfunction. However, this did not interfere with the aim of this study which was mainly designed to test the feasibility and applicability of circumferential strain estimates of the RV free wall as a surrogate of function before testing this method on patients with RV dysfunction. It should be noted that although the current study addressed intra-observer, inter-observer and inter-subject reproducibility, the scan-rescan reproducibility for the same subjects was not examined. This point is intended to be addressed in a more comprehensive future study.

It should also be noted that evaluation of regional heart function, which naturally occurs in the three dimensions, from a single circumferential plane is another limitation of the method used. Also, the imaging technique used did not correct for tissue through-plane motion that could affect the accuracy of the strain estimates. However, development of new methods for tissue slice-following [28] and motion tracking [29] could correct for this through-plane displacement.

Finally, areas with consistent low circumferential strains were found at the insertion site of the papillary muscle into the RV free wall. These areas are not likely identified in prior studies relying on imaging techniques with intrinsically lower spatial resolution. These findings direct attention to the cautious interpretation of strain estimates in light of image quality and the presence or absence of image artifacts, especially in high-field CMR systems.

## Conclusion

SENC CMR allows for rapid quantification of RV regional function with low intra- and inter-observer variabilities, which could permit accurate quantification of regional function in patients with RV dysfunction.

## Competing interests

Dr. Nael F. Osman is a founder and shareholder in Diagnosoft Inc. The terms of this arrangement have been approved by the Johns Hopkins University in accordance with its conflict of interest policies.

## Authors' contributions

AY participated in the study design and CMR exams, carried out the image and statistical analysis, and wrote and revised the manuscript. EHI assisted in the development of the CMR pulse sequence and participated in the CMR exams. GK participated in the analysis of the CMR data and in manuscript editing. MRA participated in the study design. RGW participated in the study design and critically reviewed the manuscript. NFO conceived and supervised the study and participated in its revision. All authors read and approved the final manuscript.
